# Beans (*Phaseolus* ssp.) as a Model for Understanding Crop Evolution

**DOI:** 10.3389/fpls.2017.00722

**Published:** 2017-05-08

**Authors:** Elena Bitocchi, Domenico Rau, Elisa Bellucci, Monica Rodriguez, Maria L. Murgia, Tania Gioia, Debora Santo, Laura Nanni, Giovanna Attene, Roberto Papa

**Affiliations:** ^1^Department of Agricultural, Food and Environmental Sciences, Marche Polytechnic UniversityAncona, Italy; ^2^Department of Agriculture, University of SassariSassari, Italy; ^3^School of Agricultural, Forestry, Food and Environmental Sciences, University of BasilicataPotenza, Italy

**Keywords:** domestication, genetic diversity, adaptation, population genomics, crop evolution, convergent evolution, selection signatures

## Abstract

Here, we aim to provide a comprehensive and up-to-date overview of the most significant outcomes in the literature regarding the origin of *Phaseolus* genus, the geographical distribution of the wild species, the domestication process, and the wide spread out of the centers of origin. *Phaseolus* can be considered as a unique model for the study of crop evolution, and in particular, for an understanding of the convergent phenotypic evolution that occurred under domestication. The almost unique situation that characterizes the *Phaseolus* genus is that five of its ∼70 species have been domesticated (i.e., *Phaseolus vulgaris, P. coccineus, P. dumosus, P. acutifolius*, and *P. lunatus*), and in addition, for *P. vulgaris* and *P. lunatus*, the wild forms are distributed in both Mesoamerica and South America, where at least two independent and isolated episodes of domestication occurred. Thus, at least seven independent domestication events occurred, which provides the possibility to unravel the genetic basis of the domestication process not only among species of the same genus, but also between gene pools within the same species. Along with this, other interesting features makes *Phaseolus* crops very useful in the study of evolution, including: (i) their recent divergence, and the high level of collinearity and synteny among their genomes; (ii) their different breeding systems and life history traits, from annual and autogamous, to perennial and allogamous; and (iii) their adaptation to different environments, not only in their centers of origin, but also out of the Americas, following their introduction and wide spread through different countries. In particular for *P. vulgaris* this resulted in the breaking of the spatial isolation of the Mesoamerican and Andean gene pools, which allowed spontaneous hybridization, thus increasing of the possibility of novel genotypes and phenotypes. This knowledge that is associated to the genetic resources that have been conserved *ex situ* and *in situ* represents a crucial tool in the hands of researchers, to preserve and evaluate this diversity, and at the same time, to identify the genetic basis of adaptation and to develop new improved varieties to tackle the challenges of climate change, and food security and sustainability.

## Introduction

Beans (*Phaseolus* spp.), and in particular the common bean *P. vulgaris* L., represent the most important grain legume for direct human consumption worldwide. They are a major source of highly valuable plant protein and micronutrients (Broughton et al., 2003; Vaz Patto et al., 2015), they provide health benefits that are related to their regular consumption (Messina, 2014; Bitocchi et al., 2016b), and they contribute to sustainable improvements to the environment when they are grown in agricultural rotation or with intercropping, due to their biological nitrogen fixation, their effects on the soil, and their control of weeds (Rubiales and Mikic, 2015; Bitocchi et al., 2016b). Thus, beans have a key role in the diversification and sustainable intensification of agriculture, particularly in light of the new and urgent challenges, such as climate change. However, it has to be considered that without a deep knowledge of the evolutionary history of crops, no improvements are possible, inasmuch as evolutionary studies provide breeders with information about the available genetic diversity and the genetic control of important agronomic traits related to adaptation and domestication (Bitocchi et al., 2016b).

As Charles Darwin suggested, crop domestication can be seen as a “giant experiment” to test the evolutionary hypothesis. During domestication, similar sets of traits were selected over a wide range of plant species, as the so-called domestication syndrome, which shows numerous examples of convergent phenotypic evolution. *Phaseolus* (2n = 2X = 22) is a unique example of multiple parallel and independent domestications. Indeed, not only did domestication occur in five closely related species, *Phaseolus vulgaris, P. lunatus, P. coccineus, P. dumosus* (formerly *P. polyanthus*), *P. acutifolius*, but also, different from other crop species, both *P. vulgaris* and *P. lunatus* have undergone two independent domestications. One was in Mesoamerica and the other in the Andes, which occurred during their reproductive isolation that was caused by the geographical barriers between these gene pools. Thus, considering single independent domestication events for *P. coccineus, P. dumosus* and *P. acutifolius*, and two for both *P. vulgaris* and *P. lunatus*, at least seven independent and isolated processes of domestication have occurred for *Phaseolus*.

A different question that arises is the occurrence of multiple domestications within species or within gene pools, where after domestication there was a lack of, or there were incomplete, reproductive barriers, with gene flow occurring among early domesticates, as can also be seen in for other crops (Meyer et al., 2012). In most of these cases where multiple domestications have occurred, this included gene flow between the early domesticates, while for beans, the strong geographical isolation between the gene pools guaranteed their reproductive isolation. However, this does not exclude *per se* the occurrence of multiple domestications within each gene pool of *P. lunatus* and *P. vulgaris*, and this topic is discussed later in this review. The only example that might be similar to *Phaseolus* was the domestication of rice, with the independent domestication of the *indica* and *japonica* subspecies in Asia (Vitte et al., 2004; Londo et al., 2006). However, recent studies appear to demonstrate a more complex situation for rice, which involved a single domestication with the subsequent divergence of these two subspecies (Molina et al., 2011; Choi et al., 2017).

The *Phaseolus* genus also represents a very interesting model because the divergence among these five *Phaseolus* spp. is relatively recent (2–4 Ma ago; Delgado-Salinas et al., 2006). Indeed, genomic and cytogenetic analyses have reported high levels of collinearity and synteny among the *Phaseolus* genomes (Bonifácio et al., 2012; Fonsêca and Pedrosa-Harand, 2013; Gujaria-Verma et al., 2016), which suggests conserved gene function.

The *Phaseolus* spp. have different breeding systems and life history traits, from annual and autogamous, to perennial and allogamous. This provides the opportunity to determine whether these features have direct consequences on the effects of domestication on the phenotypic and genotypic architecture of the crop plants. Another important aspect, at least for the common bean, is the complex pattern of expansion and the pathways of distribution out of the American domestication centers. This also involved several introductions from the New World that were combined with exchanges between continents, and among several countries within continents. Thus, along with a dramatic increase in the amplitude of the agro-ecological conditions for these crops, new genetic combinations have allowed new opportunities for natural and human-mediated selection that might have promoted their adaptation to specific environmental conditions.

All of these features make *Phaseolus* spp. an ideal model to study domestication and evolution. Thus, the present review offers an overview of the current knowledge of the evolutionary history of the *Phaseolus* crop species, with particular focus on *P. vulgaris* L. and on the recent outcomes relating to the genetic bases of important domestication and adaptation traits.

## Origin of the Species of the *Phaseolus* Genus

**FIGURE 1:**
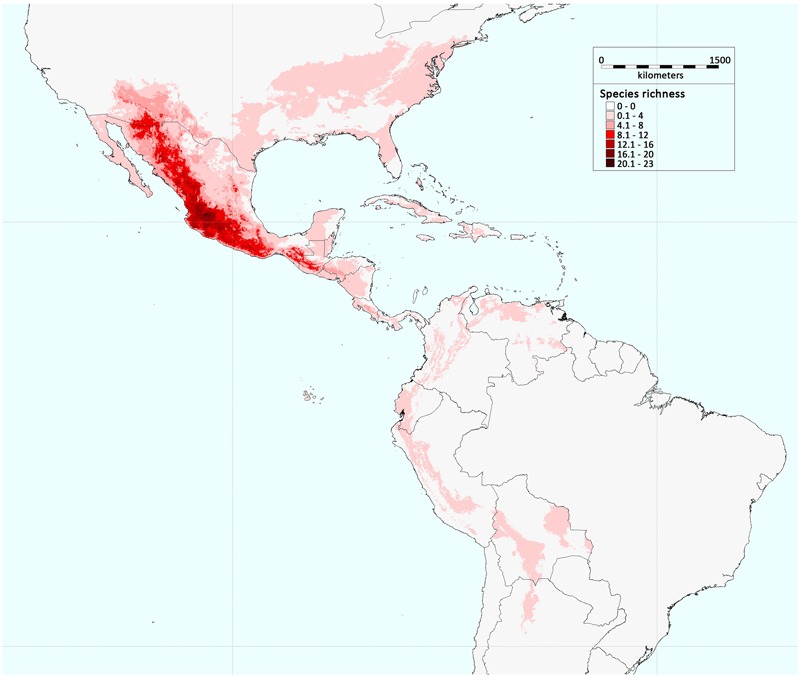
**Geographic distribution of *Phaseolus* species richness in America.** The colors in the legend indicate the number of *Phaseolus* species present in a defined geographic area (modified from Ramírez-Villegas et al., 2010; permissions for reproduction have been obtained from the copyright holders).

Among the ∼70 species that belong to the *Phaseolus* genus, most are geographically distributed in Mesoamerica (**Figure 1**), where the genus appears to have diversified within the past 4–6 Ma ago (Delgado-Salinas et al., 2006). This diversification of the different species is likely to have taken place during and after the tectonic events that led to the present-day form of Mexico (Delgado-Salinas et al., 2006), which appeared in the Late Miocene (5 Ma ago; Nieto-Samaniego et al., 1999; Alva-Valdivia et al., 2000). In particular, phylogenetic analyses have shown that the *Phaseolus* spp. can be grouped into two major sister clades: clade A, which comprises the Pauciflorus, Pedicellatus, and Tuerckheimii groups, and the weakly resolved species (i.e., *P. glabellus, P. macrolepis, P. microcarpus*, and *P. oaxacanus*); and clade B, which comprises the Filiformis, Vulgaris, Lunatus, Leptostachyus, and Polystachios groups (Delgado-Salinas et al., 2006). Thus, eight principal crown clades that show some morphological, ecological and bio-geographic distinctions characterize the *Phaseolus* genus, and their formation occurred relatively late on, with an average age of ∼2 Ma ago (Delgado-Salinas et al., 2006). The oldest group is Vulgaris, which has been dated at ∼4 Ma ago (Delgado-Salinas et al., 2006).

**FIGURE 2:**
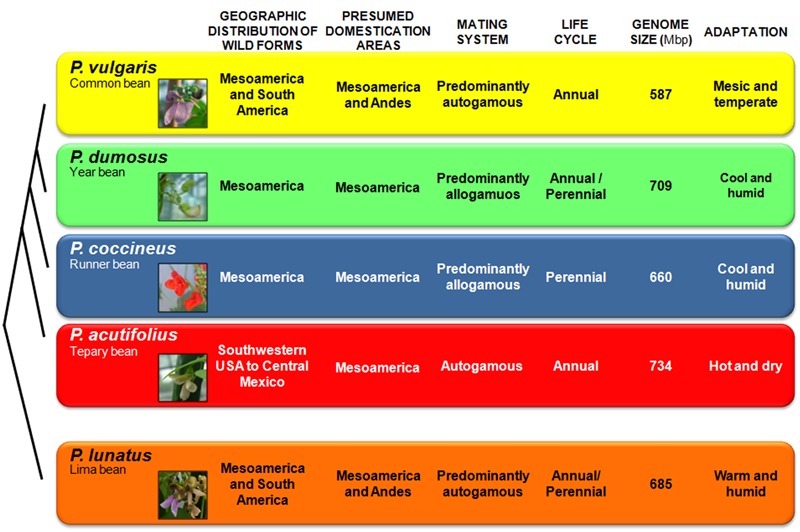
**Phylogenetic relationships of the five domesticated *Phaseolus* species, along with a comparison of their features.** These relate to the geographic distribution of the wild forms, and their presumed domestication areas, mating systems, life cycles, genome sizes and ecological adaptations. Genome size data are from the Bennett and Leitch Plant DNA *C*-values database (release 6.0, Dec. 2012, http://data.kew.org/cvalues/).

Five *Phaseolus* species were domesticated: the common bean *P. vulgaris*; the year bean *P. dumosus* Macfad.; the runner bean *P. coccineus* L.; the tepary bean *P. acutifolius* A. Gray; and the Lima bean *P. lunatus* L.. The Lima bean belongs to the Lunatus group, the formation of which has been dated at ∼2 Ma ago (Delgado-Salinas et al., 2006); it is a predominantly autogamous species that includes both annual determinate bush types and indeterminate climbers that are often perennials, due to their enlarged tap root (Baudoin, 1988; Salunkhe and Kadam, 1998) (**Figure 2**). All the other *Phaseolus* crop species (i.e., *P. vulgaris, P. dumosus, P. coccineus*, and *P. acutifolius*) belong to the Vulgaris group. In particular, *P. vulgaris, P. dumosus*, and *P. coccineus* are very closely related, and these three species are partially intercrossable, although only when *P. vulgaris* is the female parent (Mendel, 1866; Wall, 1970; Shii et al., 1982; Hucl and Scoles, 1985). This is despite their marked differences in mating systems and life cycles, as *P. vulgaris* is predominantly autogamous and annual, *P. coccineus* is predominantly allogamous and perennial, and *P. dumosus* has intermediate characteristics between *P. coccineus* and *P. vulgaris* (**Figure 2**). Analysis of sequence data of the α-amylase inhibitor gene indicated that *P. vulgaris* diverged from *P. dumosus* and *P. coccineus* ∼2 Ma ago (Gepts et al., 1999). *P. acutifolius*, is an annual species with a highly selfing reproductive system.

## Geographic Distribution, Origin and Adaptation of the Wild Forms of Domesticated *Phaseolus* Species

### Geographic Distribution and Origin

**FIGURE 3:**
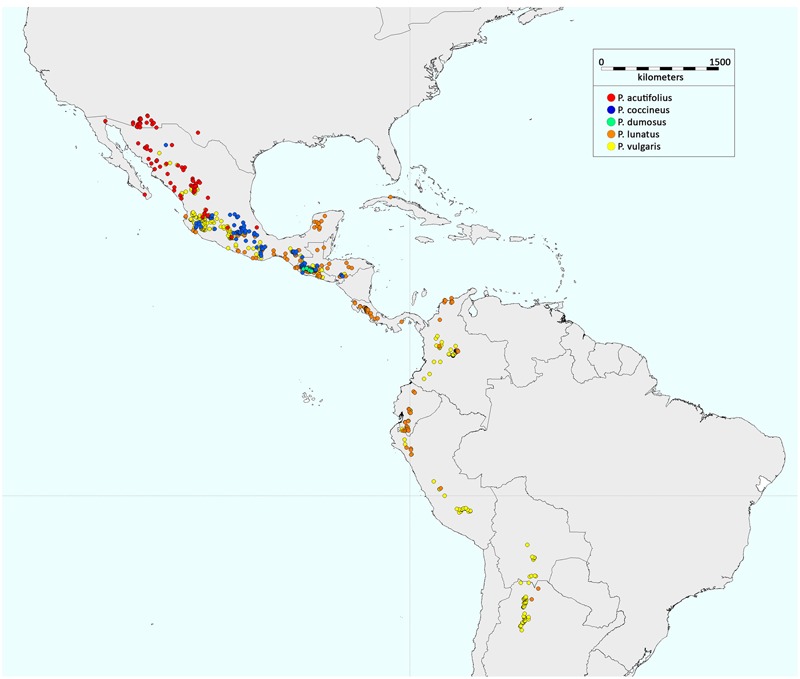
**Geographic distribution of the wild forms of the five domesticated *Phaseolus* species.** The distributions were obtained by considering all of the wild accessions with passport data in the database of the International Center for Tropical Agriculture (CIAT).

The wild forms of *P. vulgaris* and *P. lunatus* are distributed in both Mesoamerica and South America, while those of *P. dumosus, P. coccineus*, and *P. acutifolius* have a geographic distribution that is restricted to Mesoamerica (**Figure 3**). There have been numerous studies that have investigated the origin and evolution of *P. vulgaris*, which among these five domesticated *Phaseolus* spp. has the greatest economic importance. In contrast, there have been very few studies into the origins of *P. lunatus, P. acutifolius, P. coccineus*, and *P. dumosus*.

#### Phaseolus vulgaris

As proposed by Gepts (1988), a gene pool structure is identified by the observation within a biological species of strong population differentiation due to reproductive isolation, often associated to different adaptation, which can be due to geography and/or sexual incompatibility. Thus, the wild forms of the common bean, which grow from northern Mexico to north-western Argentina (Toro et al., 1990; **Figure 3**), are characterized by three eco-geographic gene pools. Two of these, the Andean and Mesoamerican, are the major gene pools of the species, and they include both wild and domesticated forms (Bitocchi et al., 2013). The third gene pool is represented by wild populations that grow in northern Peru and Ecuador, in a narrow altitudinal fringe on the western and eastern slopes of the Cordillera, a region characterized by diverse environmental conditions that differ from those on which the other wild Andean forms, including Colombian populations, grow (Debouck et al., 1993). Several studies have indicated the specific patterns of allelic frequencies and linkage disequilibrium that are characteristic of the populations from northern Peru and Ecuador (Papa and Gepts, 2003; Kwak and Gepts, 2009; Nanni et al., 2011; Bitocchi et al., 2012; Desiderio et al., 2013; Rodriguez et al., 2016; Rendón-Anaya et al., 2017). This gene pool has only been described for wild populations, with no domesticated forms ever found. Moreover, the populations from northern Peru and Ecuador are characterized by a specific phaseolin type, known as ‘Inca’ (I), which is not found in individuals outside of this geographic location (Kami et al., 1995). In particular, the findings of Kami et al. (1995) indicated that the Inca sequence of the portion of the gene that codes for the protein phaseolin is ancestral to the other types of phaseolin in individuals from the Mesoamerican and Andean major gene pools. They thus indicated northern Peru and Ecuador as the area of origin of the common bean, from where, subsequently, the species became widespread northwards (Mesoamerica) and southwards (Andes), hence leading to the formation of the two major gene pools that are characteristic of *P. vulgaris*.

**FIGURE 4:**
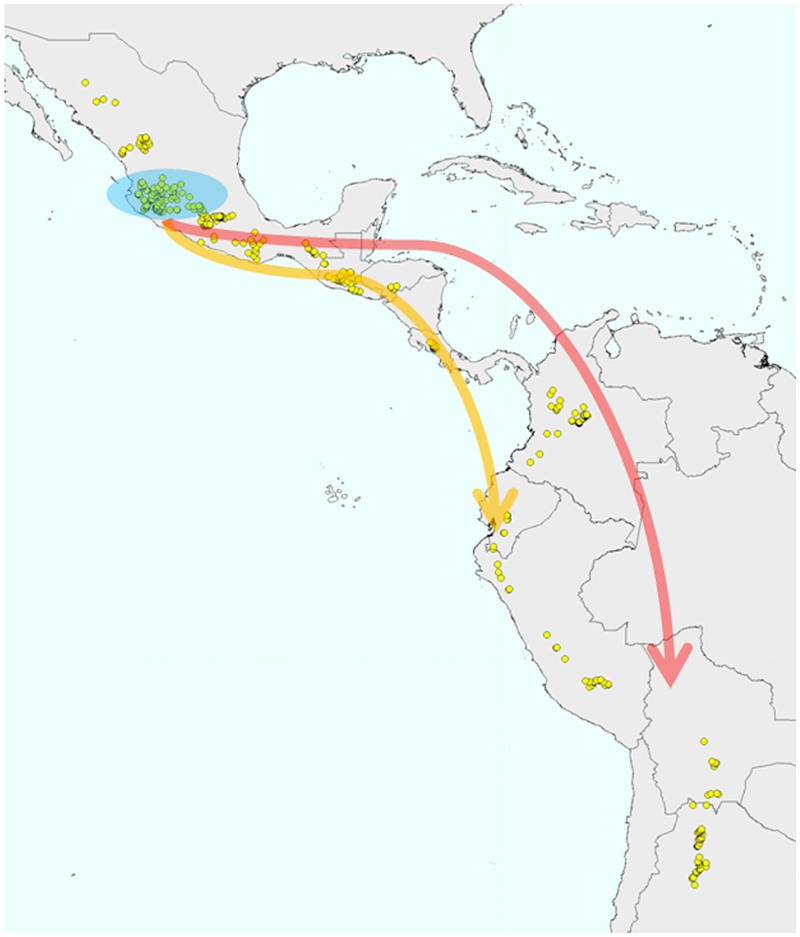
**Andean wild *P. vulgaris* populations originated from migrations of Mesoamerican wild populations that occurred prior to the domestication of the species**.

However, more recently, this evolutionary scenario was called into question by the studies of Rossi et al. (2009), and in particular, of Bitocchi et al. (2012), where their data analysis clearly indicated that both the Andean and the Inca gene pools are derived from independent introductions from Mesoamerica (**Figure 4**). In contrast to the previous studies that were based on multilocus molecular markers, Bitocchi et al. (2012) investigated the evolutionary history of the common bean using nucleotide sequences for five gene fragments. The first outcome was the clear confirmation of a bottleneck that occurred prior to domestication for the Andean gene pool. This was suggested previously by other studies (i.e., Rossi et al., 2009), although it was the lower mutation rate characteristic of nucleotide data compared to multilocus molecular markers that allowed the strong effect of the bottleneck on the genetic diversity of the Andean wild germplasm to be highlighted (i.e., a 90% reduction in diversity compared to the Mesoamerican wild gene pool; Bitocchi et al., 2012). Moreover, as sequence data from a single locus of a few 100 base pairs are less prone to recombination than multilocus molecular markers, these data allowed the identification of a strong population structure in Mesoamerica, although for the first time without any clear distinction between the Mesoamerican and Andean wild gene pools (Bitocchi et al., 2012). Indeed, the phylogenetic relationships between the different groups showed two Mesoamerican groups (from Mexico) that appeared to be more closely related, one to the northern Peru–Ecuador gene pool and the other to the Andean gene pool. This led to the conclusion that each of these gene pools from South America originated through different migrations from the Mesoamerican populations of central Mexico. This hypothesis was also supported by subsequent studies (Schmutz et al., 2014; Rendón-Anaya et al., 2017). However, Rendón-Anaya et al. (2017), differently from Bitocchi et al. (2012) and Desiderio et al. (2013), by analyzing whole genome sequencing data from 18 *P. vulgaris* accessions (eight wild and two domesticated Mesoamerican accessions; one wild and two domesticated Andean accessions and five accessions from northern Peru and Ecuador), suggested that the northern Peru and Ecuador group should be considered as a sister species on the basis of the observation of a complete separation between the northern Peru and Ecuador genotypes and a group composed by both Mesoamerican and Andean genotypes. However, from the works of Bitocchi et al. (2012) and Desiderio et al. (2013), presenting a larger sample compared to Rendón-Anaya et al. (2017), for both nuclear and chloroplast genome, wild populations from Mesoamerica closely related to the northern Peru and Ecuador group were identified, suggesting caution on the claim that the northern Peru and Ecuador group represents a different species. The whole genome sequencing analysis conducted by Schmutz et al. (2014), who estimated the divergence time between the Andean and Mesoamerican gene pools by applying demographic modeling, suggests that the wild populations in the Andes were derived from an ancestral Mesoamerican population ∼165,000 years ago. The first attempt to date the split between the Andean and Mesoamerican gene pools was represented by the study of Gepts et al. (1999), where this event was estimated to have occurred ∼500,000 years ago on the basis of the analysis of the α-amylase inhibitor and internal transcribed spacer sequence data. Mamidi et al. (2013) identified an earlier date (∼110,000 years ago) compared to that estimated by Schmutz et al. (2014). This recent divergence is in agreement with the high similarity between the genomes and with the recent observation by Vlasova et al. (2016) that most of the bean-specific gene family expansion predates the split between Mesoamerica and the Andes.

#### The Other *Phaseolus* Crop Species

The *P. lunatus* wild forms are widely distributed from central Mexico to northern Argentina (Allard, 1960; Heiser, 1965; Freytag and Debouck, 2002; **Figure 3**). Studies of the evolutionary history of *P. lunatus* have essentially been based on limited genomic data. In particular, most of these have relied on the analysis of two intergenic spacers of the chloroplast DNA (i.e., *atpB*-*rbcL, trnL*-*trnF*) and the sequence of the nuclear ribosomal 5.8S and flanking internal transcribed spacers (the ITS region) (Motta-Aldana et al., 2010; Serrano-Serrano et al., 2010, 2012; Andueza-Noh et al., 2013). Serrano-Serrano et al. (2010) analyzed these nuclear and non-coding chloroplast DNA markers in a collection of 59 wild Lima bean accessions and six related *Phaseolus* spp., three of which were of Andean distribution (i.e., *P. augusti, P. pachyrrhyzoides*, and *P. bolivianus*), with the others distributed in Mesoamerica (i.e., *P. leptostachyus, P. marechalii*, and *P. novoleonensis*). Using neighbor-joining tree analysis, they identified three divergent wild Lima bean gene pools: Ecuador and northern Peru (AI); Mexico, and mainly the area to the west and northwest of the Isthmus of Tehuantepec (MI); and Mexico, and mainly the area to the east and southeast of the Isthmus of Tehuantepec (MII). Moreover, they suggested an Andean origin for wild Lima bean, as had been reported previously (Caicedo et al., 1999; Delgado-Salinas et al., 1999; Fofana et al., 1999). Their main evidence was the close phylogenetic relationship between the wild Lima bean and the related Andean *Phaseolus* species. In agreement with the study of Delgado-Salinas et al. (2006), Serrano-Serrano et al. (2010) indicated a relatively recent origin of *P. lunatus*, to during the Pleistocene and after the major Andean orogeny, ∼2 to 5 Ma ago (Gregory-Wodzicki, 2000; Young et al., 2002). More recent studies based on the same markers and large samples of wild materials (Serrano-Serrano et al., 2012; Andueza-Noh et al., 2013) have confirmed the structure of the Mesoamerican wild populations, with the identification of the two main groups (MI, MII) with a geographic distribution that is probably related to adaptation to the different environments. Indeed, as indicated by Serrano-Serrano et al. (2012), the MI gene pool is mainly distributed in tropical dry forests over the Pacific coastal plain in Mexico at an average altitude of ∼450 m a.s.l., with a small group of accessions on the western side of the Neo-Volcanic Axis at higher altitudes (from 1,250 to 1,810 m a.s.l.), while the MII gene pool is present in the Mexican lowlands (∼550 m a.s.l.) along the Atlantic coast (Mexican gulf) and the Yucatan peninsula, and to the southeast of the Isthmus of Tehuantepec, the Caribbean, and South America.

Recently, Martínez-Castillo et al. (2014) analyzed 67 wild *P. lunatus* accessions from Mexico using 10 microsatellite (simple sequence repeats; SSR) markers. Through population structure analysis, they suggested that the genetic structure of the wild Lima bean in Mexico is more complex than previously thought, and they proposed three gene pools in Mesoamerica (MIa, MIb, and MII).

However, comparisons of the results of these studies show that there remain some disagreements, with different assignments to the diverse gene pools for the same accessions, according to the nuclear and chloroplast markers. Moreover, in some cases, the groups are not well supported statistically, and thus further studies are needed to more deeply understand the evolution of the Lima bean *P. lunatus*.

As mentioned above, the other three *Phaseolus* crop species (i.e., *P. acutifolius, P. coccineus*, and *P. dumosus*) are distributed in North and Central America. The wild forms of the tepary bean, which include *P. acutifolius* var. *acutifolius* and *P. acutifolius* var. *tenuifolius*, grow in the region from Central Mexico to southwestern USA (Blair et al., 2002; **Figure 3**). Indeed, this species is believed to have originated within this geographic range (Freeman, 1912, 1913; Nabhan and Felger, 1978; Manshardt and Waines, 1983).

The wild forms of the runner bean *P. coccineus* are distributed from Chihuahua (Mexico) to Panama (Delgado Salinas, 1988). The few archeological remains available (Delgado Salinas, 1988; Kaplan and Lynch, 1999) also indicate this area as the center of origin of *P. coccineus*.

For the wild form of the year bean *P. dumosus*, the geographic distribution is centered on a very narrow area in Guatemala, and this is where it has been suggested that *P. dumosus* originated (Schmit and Debouck, 1991).

### Adaptation

**FIGURE 5:**
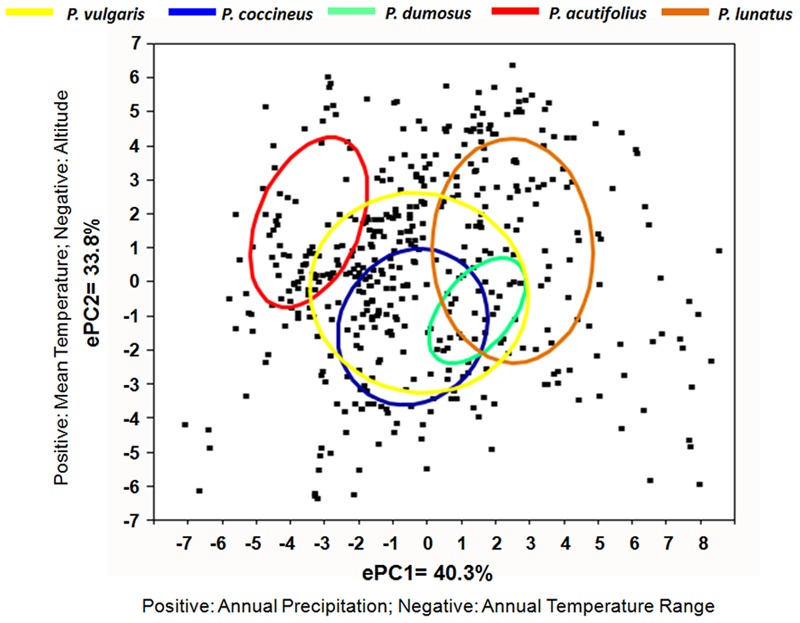
**Relationships among wild accessions of *Phaseolus* crop species as a function of the first two ecological principal components (ePC1, ePC2).** The 50% density ellipses are calculated for each species.

Genetic diversity is not uniformly distributed throughout the variety of climatic and environmental conditions under which a species grows. Consequently, it depends not only on demographic processes (e.g., genetic drift), but also on adaptation, in terms of the ability to cope with specific environmental conditions. These conditions can include extremes of cold and heat, lack or excess of water, different light intensities and duration, and diverse soil conditions and pest and disease pressures. As indicated above, the wild forms of *Phaseolus* crop species grow under a variety of different environmental and climatic conditions, and their geographic distribution mirrors their diverse patterns of adaptation to the different ecological niches, as well as their life histories and reproductive systems (**Figure 2**). *P. vulgaris* is adapted to warmer temperatures (mesic and temperate soil temperatures) at lower altitudes, with a rainfall of ∼1,100 mm/year (Supplementary Table S1). *P. coccineus* is found in more humid environments, at cooler temperatures, and at higher altitudes, while *P. dumosus* is characterized by intermediate adaptation. *P. acutifolius* is a drought-tolerant species that originated in warmer and more arid environments (e.g., it is grown in the arid lands of Mexico and in southeast USA). Finally, *P. lunatus* is particularly suited to low-altitude humid and sub-humid climates, as well as warm temperate zones (**Figure 2** and Supplementary Table S1). These observation are confirmed by principal component analysis (**Figure 5**). This was calculated from the data on 20 ecological variables (Supplementary Table S1) using DIVA-GIS 7.5^1^, as inferred from the geographical coordinates of the collection sites of the wild accessions for the passport data present in the database of the International Centre for Tropical Agriculture (CIAT). Moreover, as can be seen in **Figure 5**, among these five species, *P. vulgaris* shows the widest ecological adaptation.

**FIGURE 6:**
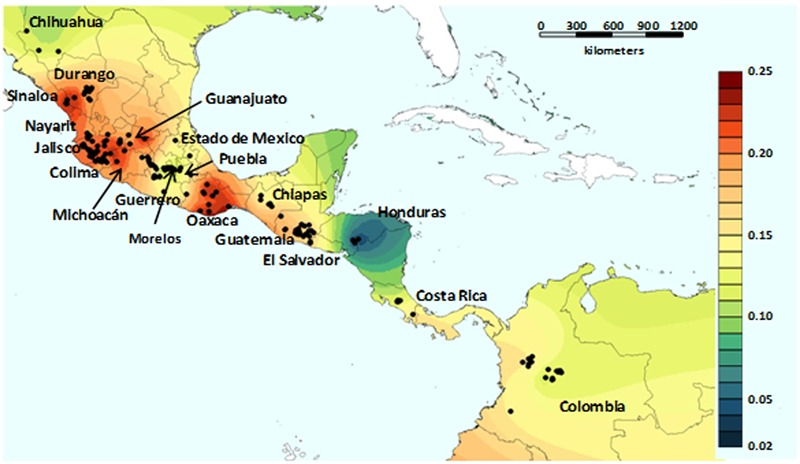
**Genetic heat-map generated by diversity interpolation based on an individual-centerd approach.** The genetic topography of *P. vulgaris* varies from low (blue) to high (red) diversity levels, which indicate the generally higher levels of diversity in Mesoamerica with respect to the Andes (from Rodriguez et al., 2016; permissions for reproduction have been obtained from the copyright holders).

There is little in the literature that is aimed at highlighting the genetic basis of adaptation, especially for the wild forms of these crop species, although a few recent studies have been focused on *P. vulgaris*. Rodriguez et al. (2016) carried out a study on the environmental adaptation of wild *P. vulgaris*. They investigated the role of demographic processes (e.g., genetic drift) and selection for adaptation in the shaping of the current genetic structure of wild *P. vulgaris*. This analysis was based on 131 single nucleotide polymorphisms (SNPs) on a sample of 417 wild *P. vulgaris* accessions that are representative of the geographic distribution of the wild forms of the species. They first investigated the spatial distribution of the genetic diversity of the wild forms of *P. vulgaris*, using a landscape genetics approach that was based on an individual centered analysis to avoid sampling bias (**Figure 6**). Briefly, they delineated a circular neighborhood of 100-km radius around each georeferenced accession, and the accessions falling within each neighborhood were used to calculate the relative unbiased gene diversity, He (Nei, 1978). The correlation between He and the neighborhood size was not significant (*r* = 0.040, *n* = 299, *P* = 0.482), and the mean size of each neighborhood was 40.6 individuals and 83.3% of the neighborhoods included >10 individuals (Rodriguez et al., 2016). By using this approach, they observed high genetic diversity across Mexico, from the state of Oaxaca to Durango, with a depression in the genetic diversity in an area that lies approximately across the regions of Guerrero, Morelos, Puebla and Estado de Mexico. Possible explanations for the reduced diversity of this area in Mexico include natural selection due to an environment that is too arid for *P. vulgaris*, or a genetic bottleneck caused by the volcanic activities that were frequent in this area in ancient times (Márquez et al., 1999; Siebe, 2000; Siebe et al., 2004; Plunket and Uruñuela, 2012). Low diversity was also found in Guatemala, Costa Rica and Colombia, and particularly in the Honduras (**Figure 6**).

The approach applied by Rodriguez et al. (2016) also highlighted local adaptation at the continental level. The rationale behind the study was that the use of spatial data in combination with genetic diversity data would allow discrimination between the effects of geography and ecology; i.e., demographic processes vs. selection (Bradburd et al., 2013; Wang et al., 2013; Guillot et al., 2014; Kraft et al., 2014). As evidence of the effectiveness of this approach, it was possible to disentangle the effects of geography from ecology in the shaping of the genetic patterns observed, and correlations between markers and ecological variables were detected. By scanning the SNP markers used for the analyses, 26 loci (19.8%) were identified as under signatures of selection, seven of which (5.3%) showed strong probability levels. Although the proportion of loci under selection might be overestimated, as they were chosen among genes that were putatively involved in adaptation, different loci were found to have compatible functions with adaptation features, such as cold acclimation, chilling susceptibility, and mechanisms related to drought stress. Moreover, as well as the well-delineated genetic groups in the Mesoamerican gene pool, they demonstrated global structures for both the neutral loci and the loci under selection. Overall, these data suggest that the origin of the geographic structures might be the outcome of the expansion of the species and gene flow, including crop-to-wild introgression (Papa and Gepts, 2003; Papa et al., 2005), as was also recently confirmed by Rendón-Anaya et al. (2017) using whole genome sequencing analysis. Nonetheless, the adaptation to different environmental conditions might have led to the present population structure, with the subsequent limited long-range gene flow and divergent selection along an ecological cline of variation.

## Domestication of *Phaseolus* spp.

In the context of domestication, the *Phaseolus* spp. represent a unique model, where these five closely related species provide a unique example of multiple parallel domestication events, which allow to investigate this evolutionary process as a kind of replicate experiment. Two of these species (i.e., *P. vulgaris* and *P. lunatus*) have gone through at least two isolated and independent domestication processes, due to the distribution of the wild forms. For the common bean *P. vulgaris*, two independent events in the Americas (in Mesoamerica and the Andes) have been documented in several studies (for review, see Bellucci et al., 2014b), where the two major domesticated gene pools originated (Bitocchi et al., 2013). A similar scenario has been observed for *P. lunatus*, where at least two independent domestication events have been suggested. One of these was in the Andes, which appears to have given rise to the large-seeded landraces collectively known as the ‘big Lima’ cultivars (Motta-Aldana et al., 2010). The other was in Mesoamerica, which appears to have given rise to the great variety of small-seeded Mesoamerican landraces (Motta-Aldana et al., 2010; Serrano-Serrano et al., 2012).

Not so much is known for the other three domesticated *Phaseolus* spp.. Through an analysis of a small set of wild and domesticated *P. coccineus* accessions and using chloroplast SSRs, Angioi et al. (2009a) identified two different wild genetic groups that paralleled the differentiation between two groups of the domesticated accessions, which suggested multiple domestication events for *P. coccineus* in Mesoamerica. A single domestication event was suggested for *P. dumosus* by Schmit and Debouck (1991) on the basis of seed protein data that were analyzed on a sample of 163 wild and domesticated accessions, as also for *P. acutifolius*, through studies based on phaseolin (Schinkel and Gepts, 1988), isoenzymes (Garvin and Weeden, 1994), SSRs (Blair et al., 2012), and SNPs (Gujaria-Verma et al., 2016). In particular, Gujaria-Verma et al. (2016) analyzed 645 SNPs markers on a wide sample that included both wild and domesticated *P. acutifolius* accessions, and they reported that domesticated tepary beans formed a tightly linked cluster that was subdivided into two major groups based on their eco-geographical origin (Central America and USA/Mexico). Moreover, the domesticated accessions were clearly separated from the wild, which suggested that it was likely that there had been an early domestication event that was followed by separation based on regions.

Overall, for the *Phaseolus* species, at least seven independent domestication events have occurred, with examples seen of both multiple and single domestication processes.

### Geographic Areas of *Phaseolus* ssp. Domestication

One of the major issues of evolutionary studies that are focused on the domestication of plant species is to identify the geographic areas where they were domesticated (Harlan, 1975).

#### Phaseolus vulgaris

Considering the process of domestication within each gene pool, after the long debate for *P. vulgaris* on this issue (for review, see Bellucci et al., 2014b), recent studies have indicated that a single domestication event occurred within each gene pool (Mamidi et al., 2011; Nanni et al., 2011; Bitocchi et al., 2013).

For the Mesoamerican gene pool, several important crops were domesticated in Mexico, including maize, squash and common bean, as has been well documented in different studies based on both archeological and molecular data (for review, see Bitocchi et al., 2013). Recently, based on SSR data, Kwak et al. (2009) suggested the Rio Lerma–Rio Grande de Santiago basin in western-central Mexico as the putative geographic area where the common bean *P. vulgaris* was domesticated. The domestication area they suggested for the common bean did not overlap with that indicated for maize (central Balsas River drainage; Matsuoka et al., 2002; van Heerwaarden et al., 2011). Thus Kwak et al. (2009) proposed that maize and the common bean were probably domesticated in different regions and were instead reunited later in a single cropping system: the milpa, the cropping system that forms the basis of the Mesoamerican traditional agriculture based on the intercropping of maize (*Zea mays* L.), the common bean, and squash (*Cucurbita* spp.). Bitocchi et al. (2013) used nucleotide data to suggest a different location for the domestication of the common bean: Oaxaca Valley, in the south of Mexico. This area does not coincide with that proposed by Kwak et al. (2009) for the common bean and that of maize (Matsuoka et al., 2002); however, it overlaps with one of the first areas in the spread of maize through human migration, along the Mexican rivers (Zizumbo-Villarreal and Colunga-GarcíaMarín, 2010).

The pinpointing of the geographic area where domestication took place for the common bean in the Andes has been more difficult, due to the low diversity characteristic of this germplasm. However, different studies have tried to identify the geographic area for this domestication. Chacón et al. (2005) analyzed the polymorphism at the level of the chloroplast DNA for a wide sample of common bean accessions from South America, and they suggested that central-southern Peru was the cradle of domestication of the Andean gene pool. Beebe et al. (2001) used amplified fragment length polymorphisms (AFLPs) to define a strict relationship between the domesticated forms and the wild beans from eastern Bolivia and northern Argentina, and suggested this location as the putative area of domestication, which was also supported recently by the study of Bitocchi et al. (2013).

More recently, on the basis of SNP data, the locations proposed by Bitocchi et al. (2013) for both the Mesoamerican and Andean gene pools were confirmed by the study of Rodriguez et al. (2016). Here, Rodriguez et al. (2016) integrated the results obtained from spatial, phenotypic and molecular data with those from different disciplines, including archeological and glotto-chronological data, to pinpoint the domestication sites in Mesoamerica and the Andes. The low genetic distances between the wild forms and the domesticated forms indicated these as genetic groups located in the Oaxaca Valley in Mesoamerica and in a region from northern Argentina to southern Bolivia in the Andes, respectively. Consistent with these data, previous archeological data have indicated the early occurrence of domestication in these areas (Tarrago, 1980; Kaplan and Lynch, 1999). Recent glotto-chronological studies have also supported these conclusions, as within these areas the homeland sites of proto-languages for which ancient bean words were reconstructed showed times that are compatible with the domestication of *P. vulgaris* (Brown et al., 2014). We expect that future studies will better refine the areas of domestication. For this purpose will be very important also to conduct appropriate explorations. Indeed, as shown by Zizumbo-Villarreal et al. (2009) additional wild bean populations, not yet included in germplasm banks, can still be identified.

#### The Other *Phaseolus* Crop Species

Similar to the common bean, the Lima bean *P. lunatus* has been domesticated at least twice in the Americas: once in the Andean region, and at another time in Mesoamerica. However, further studies are needed to investigate the domestication process of this species more deeply. Indeed, this still appears unclear especially for the Mesoamerican gene pool, where there remains open debate concerning its single or multiple domestication (Motta-Aldana et al., 2010; Serrano-Serrano et al., 2012; Andueza-Noh et al., 2013).

Motta-Aldana et al. (2010) analyzed chloroplast DNA and ITS polymorphisms in a sample of wild and landrace accessions of *P. lunatus*, through which they suggested one domestication event in the Andes of northwestern Peru and southern Ecuador, and a second in central-western Mexico, which they indicated as more likely to be in the area to the north and northwest of the Isthmus of Tehuantepec. Consistent with this study by Motta-Aldana et al. (2010), in their analysis of ITS data on a large sample of Mesoamerican wild and domesticated *P. lunatus*, Serrano-Serrano et al. (2012) proposed a single event of domestication in Mesoamerica. As indicated above, they showed evidence for two wild Mesoamerican gene pools with mostly contrasting geographic distributions. In their cluster analysis, all of the Mesoamerican landraces clustered together with the wild accessions from the MI gene pool, which is characteristic of central-western Mexico. This suggests a unique domestication event in an area of the states of Nayarit–Jalisco or Guerrero–Oaxaca, and not on the Peninsula of Yucatan, where *P. lunatus* is currently widespread and diverse.

Andueza-Noh et al. (2013) used two intergenic spacers of chloroplast DNA to confirm these Mesoamerican and Andean gene pools for *P. lunatus* (Gutiérrez-Salgado et al., 1995; Maquet et al., 1997; Lioi et al., 1998; Fofana et al., 2001; Motta-Aldana et al., 2010), and the two genetically and geographically distinct groups within the Mesoamerican gene pool (MI, MII; Motta-Aldana et al., 2010; Serrano-Serrano et al., 2010, 2012). They pinpointed the domestication area for the Andean gene pool as mid-altitude western valleys between Peru and Ecuador in South America, as had already been suggested (Gutiérrez-Salgado et al., 1995; Fofana et al., 2001; Motta-Aldana et al., 2010). However, in contrast to the previous studies, Andueza-Noh et al. (2013, 2015) indicated multiple origins of domestication in Mesoamerica for *P. lunatus*. For the MI group, they indicated western-central Mexico as the domestication area, while they proposed a more restricted geographic area between Guatemala and Costa Rica for the MII group. However, they were aware that more studies involving more comprehensive geographic and genomic sampling are needed to define how the domestication processes and gene flow have shaped the current genetic structure of *P. lunatus* landraces.

For the other three *Phaseolus* crops (i.e., *P. acutifolius, P. coccineus*, and *P. dumosus*), nothing much is known about their domestication or where this process might have taken place. There have been very few studies on *P. acutifolius* and *P. coccineus*. For the tepary bean *P. acutifolius*, early studies based on phaseolin and isozyme analysis highlighted the controversy over the number of domestication events. Here, some studies proposed two domestication events in the northern and southern parts of the range (Manshardt and Waines, 1983), and others suggested a single origin but different locations for the domestication, as either in the Mexican state of Durango (Schinkel and Gepts, 1988) or the states of Sinaloa or Jalisco (Garvin and Weeden, 1994). The more recent study of Blair et al. (2012) was based on SSR data on a wide sample of wild and domesticated *P. acutifolius* accessions from its area of distribution. They indicated, as mentioned above, that a single domestication event was likely, and that the cultivars were most closely related to *P. acutifolius* var. *acutifolius* accessions from Sinaloa and northern Mexico.

*Phaseolus coccineus* is native to Mexico, Guatemala and Honduras (Delgado Salinas, 1988), and the wild forms are probably not all ancestral to the cultivated form. However, the area(s) where the domestication of *P. coccineus* took place are still not known. Spataro et al. (2011) used SSR data with a collection of wild and domesticated accessions, and they showed that most of the Mesoamerican landraces they examined closely resembled wild genotypes from Guatemala and Honduras, while only a few resembled wild Mexican forms. This would suggest that *P. coccineus* domestication either took place in that area, or that two domestication events took place (in Guatemala–Honduras and Mexico, separately) followed by extensive hybridisation with the cultivated forms from Guatemala and Honduras.

The distribution of wild *P. dumosus* (the year bean) is extremely narrow on the basis of findings to date, and it appears to be concentrated only in central southwestern Guatemala. Schmit and Debouck (1991) used phaseolin data together with information on vernacular names, and they reported that there is a single gene pool that was domesticated from a wild ancestor that is still present in Guatemala. Thus, they indicated a single domestication in Guatemala, and subsequent diffusion toward the humid highlands of Chiapas, Oaxaca, Puebla and Veracruz in Mexico, and toward Costa Rica and the northern Andes.

### Domestication Bottleneck

The population genetics model of domestication predicts a reduction in diversity and increased divergence between wild and domesticated populations, due to demographic factors that affect the whole genome, and because of selection at target loci. Several interesting insights can be revealed by comparisons between different species (Glémin and Bataillon, 2009). There are many examples in the literature that have used different molecular markers and nucleotide data to show the reduction in genetic diversity of crop species compared with their wild progenitors (for review, see Bitocchi et al., 2013). Allogamous species, such as maize (*Z. mays*), are generally characterized by lower genetic bottleneck effects compared to autogamous species, such as the common bean *P. vulgaris*, even if other factors can have relevant roles, such as the life history (Bitocchi et al., 2013). Resequencing data have confirmed that in autogamous species, such as soybean (*Glycine max*) and rice (*Oryza sativa*, variety *japonica*) (Lam et al., 2010; Xu et al., 2012), reductions in diversity have arisen due to domestication, as also reported for silkworm and for mammalian species (Xia et al., 2009; vonHoldt et al., 2010; Lippold et al., 2011).

#### Phaseolus vulgaris

For the common bean *P. vulgaris*, different studies have clearly identified a bottleneck due to domestication in both the Mesoamerican and Andean gene pools (e.g., Papa et al., 2005; Kwak and Gepts, 2009; Rossi et al., 2009; Mamidi et al., 2011; Nanni et al., 2011; Bitocchi et al., 2013, 2016a; Bellucci et al., 2014a). However, the reduction in diversity in the domesticated forms compared to the wild forms was greater in Mesoamerica compared to the Andes. Indeed, Bitocchi et al. (2013) reported that this loss of diversity was threefold greater for Mesoamerica compared to the Andes, and this was explained as the result of the bottleneck that occurred before domestication in the Andes. This thus strongly impoverished the genetic variability of the Andean wild germplasm, which led to a minor effect of the subsequent domestication bottleneck (i.e., sequential bottleneck). These outcomes demonstrate that the understanding of the level and the structure of genetic diversity of a species needs to be accompanied by a close appraisal of its evolutionary history.

Bellucci et al. (2014a) exploited next-generation sequencing technologies to analyze changes at the transcriptome level in *P. vulgaris* accessions from Mesoamerica, to investigate the domestication process in this gene pool more deeply. They used RNA sequencing technology and *de novo* transcriptome assembly to compare representative sets of wild and domesticated accessions of the common bean from Mesoamerica, and they reported the profound effects that domestication imposed on the genome variation and gene expression patterns of the common bean. Indeed, they showed that in addition to reduced nucleotide variation, the domesticated common bean showed reduced gene expression diversity, while in maize, the same reduction was not seen in parallel with reduced effects of domestication for nucleotide diversity (Hufford et al., 2012; Swanson-Wagner et al., 2012). The expressed genomic regions lost half of the wild-bean nucleotide diversity during the domestication in Mesoamerica, and in parallel, the effects of domestication significantly decreased the diversity of gene expression (by 18%). For the first time, this demonstrated that loss of genetic variation has direct genome-wide phenotypic consequences on transcriptome diversity. The contigs identified as differentially expressed (in the comparison of domesticated vs. wild) were mostly down-regulated in the domesticated forms (by 74%). This indicated loss-of-function mutations (which are relatively frequent compared to gain-of-function changes) as a largely available source of variation that supports selection during rapid environmental changes (Olson, 1999). Such was the case for the transition from the wild to the cultivated agro-ecosystems. In support of this, as first noted by Darwin (1859), in domesticated plants, the domestication traits have a recessive genetic nature (Lester, 1989).

In addition to the case of differentially expressed genes, the genome-wide gene expression reported by Bellucci et al. (2014a) for the domesticated common bean *P. vulgaris* was on average lower than for the wild. They interpreted this result as the accumulation of slightly deleterious mutations due to hitchhiking (mostly loss-of-function, or with reduced expression) in *P. vulgaris*, and considered this as the ‘cost of domestication.’ This accumulation of loss-of-function (or reduced expression) mutations might also have been due to reduced effective recombination, which would have increased the frequency of deleterious mutations in the domesticated pool, and have had a negative influence on the fitness, as was suggested in rice (Lu et al., 2006).

#### The Other *Phaseolus* Crop Species

In their analysis of chloroplast DNA and ITS polymorphisms in a sample of wild and landrace accessions of *P. lunatus* (the Lima bean), Motta-Aldana et al. (2010) observed a severe reduction in genetic diversity because of domestication in both the Mesoamerican and Andean gene pools (the MI wild accessions were used for co-mutations); in particular, the loss of diversity appeared stronger according to chloroplast DNA data (100%, 92.1%, for the Mesoamerican and Andean gene pools, respectively) than for ITS data (46.6%, 58.5%, respectively). This was confirmed for the Mesoamerican gene pool by Serrano-Serrano et al. (2012) and Andueza-Noh et al. (2013, 2015), through analysis of two intergenic spacers of chloroplast DNA (loss of diversity, 60.83%), SSR markers (loss of diversity, 44%), and the ITS region of ribosomal DNA (loss of diversity, 53%).

A bottleneck of domestication was also seen for *P. acutifolius*. Genetic diversity within the domesticated forms of the tepary bean is low, as has been shown by studies of phaseolin patterns (Schinkel and Gepts, 1988), isozymes (Schinkel and Gepts, 1989; Garvin and Weeden, 1994), AFLPs (Muñoz et al., 2006), and SSR markers (Blair et al., 2012). Considering the year bean *P. dumosus*, Schmit and Debouck (1991) analyzed phaseolin data and showed that the wild ancestral forms in central Guatemala show the highest diversity.

A different scenario has been reported in the few studies on domestication of the runner bean *P. coccineus*. Escalante et al. (1994) indicated that the domestication process did not erode the genetic diversity of *P. coccineus*, and that the similar levels of genetic variation among the wild and cultivated materials were mainly due to the high gene flow between these two forms. This result was confirmed by Spataro et al. (2011) through an analysis of SSR data on a sample of wild and domesticated runner bean accessions.

### Signatures of Selection during Domestication

Identification of the genes involved in the domestication process and knowledge of the regions of the genome where those genes are located and of the proportion of the genome affected by domestication are key to better exploitation of the diversity present in the wild relatives, and to enhance the achievements in breeding and crop improvement (Tanksley and McCouch, 1997; McCouch, 2004). As proposed by Cavalli-Sforza (1966) and Lewontin and Krakauer (1973), the identification of loci involved in adaptive processes can be obtained from population genetics expectations that predict that while drift has a homogeneous effect over the genome, selection is acting only for target loci and related linked loci due to the lack of recombination (hitchhiking). Thus, selected (and linked loci) are expected to depart from neutral expectation of diversity and divergence parameters. Moreover, as proposed by Papa et al. (2005), in gene flow between crops and wild forms, aberrant patterns of divergence and diversity can also be determined by the combined actions of asymmetric migration (Papa and Gepts, 2003) and selection at target loci.

Papa et al. (2007) used 2,506 AFLPs for a whole genome scan for the signature of selection due to domestication, and they estimated that about 16% of the genome of the common bean *P. vulgaris* appeared to be under the effects of selection. Bellucci et al. (2014a) used RNA sequencing technology, and after simulating the demographic dynamics during domestication, they reported that 9% of the genes were actively selected during domestication in Mesoamerica. Furthermore, in these contigs, selection induced a further reduction in the diversity of gene expression (by 26%), and was associated with a fivefold enrichment of the differentially expressed genes.

Bellucci et al. (2014a) also carried out a survey on the function of a subset of contigs that are putatively under selection, to determine whether they are known to be associated with the domestication process in other species, using either direct experimentation or through their function. Interestingly, among the genes putatively under selection that showed greater genetic diversity in the wild compared with the domesticated form, they found sequence homologs to: (i) genes that are involved in ‘light’ response pathway; e.g., GIGANTEA (GI), which has a pivotal role in the photoperiodic response (Mizoguchi et al., 2005; Hecht et al., 2007; Kim et al., 2012); (ii) genes that are pivotal to ensure correct hormonal perception, transport or biosynthesis; (iii) genes that are involved in seed development and traits; and (iv) genes that are involved in responses to environmental stress.

**FIGURE 7:**
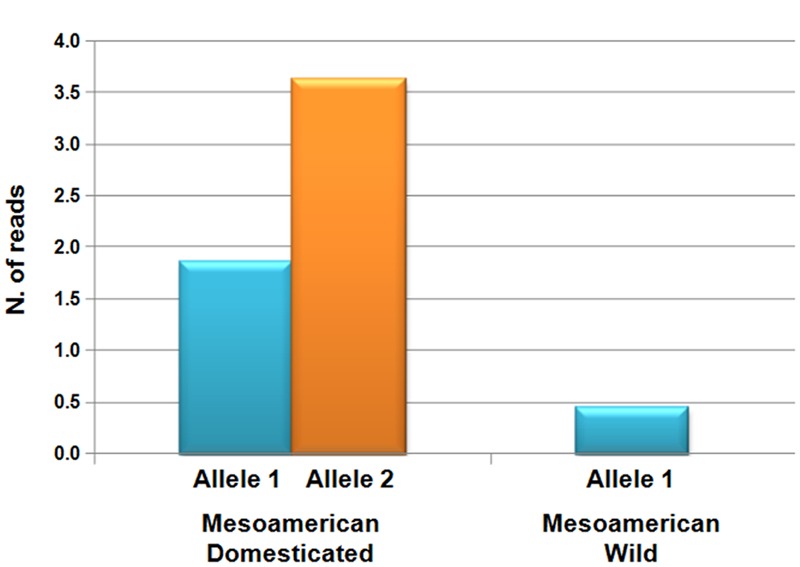
**Levels of expression (normalized numbers of reads) for the two different alleles of the *KUP6* (*K*^+^*uptake transporter6*) gene, which has been identified as under selection during common bean domestication in Mesoamerica, for wild and domesticated populations (Bellucci et al., 2014b).** The wild genotypes carried only Allele 1, while each of the two allele were present in the domesticated population.

Another interesting example is the homolog of *YABBY5* (*YAB5*), which is a transcription factor that is implicated in the regulation of seed shattering in cereal species, including sorghum (*Sorghum bicolor*), rice and maize (Lin et al., 2012). In most cases, Bellucci et al. (2014a) found evidence of positive selection associated with domestication, but in a few cases, this selection had increased the nucleotide diversity in the domesticated pool at a target locus associated with abiotic stress responses, flowering time, and morphology. In particular, for 2.8% of the genes putatively identified to be under the effect of selection by Bellucci et al. (2014a), there was no diversity in the wild forms, while there was diversity in the domesticated. They explained this as due to novel mutations (or standing variations) that were selected because of the crop expansion into new environments (diversifying selection) with unexpected biotic and abiotic stress, or because of selection for traits that improved the use of the plant organs by humans (de Alencar Figueiredo et al., 2008). An interesting example was given by the functional analysis of the drought- and growth-related (Osakabe et al., 2013) *KUP6* (*K*^+^ *uptake transporter6*) gene, where this was significantly overexpressed in the domesticated compared to the wild (**Figure 7**), as if domestication had also increased the functional diversity of selected genes in addition to the increased nucleotide diversity.

Schmutz et al. (2014) also investigated whether their candidate genes were implicated in important domestication traits, such as flowering time and seed size. A total of 38 flowering genes were identified in the Mesoamerican and Andean candidate lists, while another subset of 15 genes was found to be associated with seed size in genome-wide association studies, and 11 genes contained SNPs that were associated with seed weight.

Recently, Bitocchi et al. (2016a) analyzed nucleotide sequences from a set of 49 gene fragments from a sample of 39 wild and domesticated Mesoamerican accessions of *P. vulgaris*. In this study, they applied the same approach as Bellucci et al. (2014a), and they identified several loci that showed signatures of selection during common bean domestication in Mesoamerica. In particular, they had the possibility to see if their candidates were detected as outliers also in other studies of varying sizes, data types, and methodologies (Bellucci et al., 2014a; Schmutz et al., 2014; Rodriguez et al., 2016). They thus obtained independent evidence that four genes (i.e., *AN-Pv33, AN-Pv69, AN-DNAJ*, and *Leg223*) were targets of directional selection during common bean domestication. The gene function investigation for these genes highlighted that they are involved in plant resistance tolerance to biotic and abiotic stresses, such as heat, drought, and salinity. Moreover, another important outcome of Bitocchi et al. (2016a) was related to the observed excess of non-synonymous mutations in the domesticated germplasm. In particular, they observed a significantly higher frequency of polymorphisms in the coding regions compared to non-coding regions only in the domesticated beans. These mutations were mostly non-synonymous and were recently derived mutations present in genes related to responses to biotic and abiotic stresses. These data cannot be fully explained by the cost of domestication alone, but support a scenario where new functional mutations were selected for adaptation during domestication, showing that domestication also increased the functional diversity at target loci that enable the domesticated forms to successfully compete during the expansion and adaptation to new agro-ecological growing conditions.

### Phenotypic Convergent Evolution

The key aspect of domestication is the convergent phenotypic evolution that is associated with the adaptation to a novel agro-ecosystem, and to human needs. For instance, most domesticated animals were selected to maximize the production of useful products (e.g., meat, milk, and wool) and for their docile behavior, while crops were selected for the size of the plant organs used by humans (e.g., seeds and fruit) and for reduced, or lack of, seed dispersal. For these reasons, domestication provides us with a unique tool to understand the process of adaptation, to test evolutionary hypotheses, and to identify the molecular basis of phenotypic diversity. Several interesting insights can be revealed by comparisons among different species (Glémin and Bataillon, 2009), where, for instance, the population genetics model of domestication predicts a reduction in diversity and increased divergence between wild and domesticated populations due to demographic factors that affect the whole genome and because of selection at target loci.

In this regard, an example is given by the study of Nanni et al. (2011), in which wild and domesticated *P. vulgaris* accessions were analyzed by sequencing a genomic region of ∼1,200 bp (*PvSHP*1) that is homologous to *SHATTERPROOF*-1 (*SHP*1), a gene involved in the control of fruit shattering in *Arabidopsis thaliana* (Liljegren et al., 2000). The loss of fruit shattering has been under selection in most seed crops, to facilitate seed harvesting (Purugganan and Fuller, 2009), while in wild plants, this feature is a fundamental trait enabling seed dispersal. Expressed sequences that correspond to *SHP*1 have also been identified in other species, such as, tomato, where it was indicated as having an important role in the regulation of both fleshy fruit expansion and the ripening process (Vrebalov et al., 2009), which are together necessary to promote seed dispersal of fleshy fruit. In legumes, sequences orthologous to *Arabidopsis SHP*1 have been identified in *Medicago*, pea and soybean (Hecht et al., 2005). Nanni et al. (2011) mapped *PvSHP*1 on linkage group Pv06 of the common bean genome, and showed that it did not co-segregate with the *St* locus, which is responsible for the presence or absence of pod string, and was mapped on linkage group Pv02 using a domesticated (Midas) × wild (G12873) RIL population by Koinange et al. (1996). These results suggested that *PvSHP*1 is not responsible for the observed phenotypic variation in *P. vulgaris* for fruit shattering. Similar results were found by Gioia et al. (2013b), who sequenced and mapped *PvIND* on the common bean genome, a sequence that is homologous to the *INDEHISCENT* gene (*IND*), which is the primary factory required for silique shattering in *A. thaliana. PvIND* mapped near the *St* locus; however, the lack of complete co-segregation between *PvIND* and *St* and the lack of polymorphisms at the *PvIND* locus correlated with the dehiscent/ indehiscent phenotype suggested that *PvIND* is not directly involved in pod shattering and is not the gene underlying the *St* locus (Gioia et al., 2013b).

Recently, Murgia et al. (2017) developed a phenotyping approach in *P. vulgaris* to evaluate the shattering syndrome in a segregating population. This is a promising approach for the identification of genetic factors that control the shattering trait in the common bean, and it will greatly facilitate comparative studies among legume crops, and also gene tagging.

Within the same species, the study of Schmutz et al. (2014) represents the first example of the possibility to investigate convergent evolution between the two gene pools of the common bean *P. vulgaris*. Indeed, a comparison of the results of selection in the two gene pools, in which independent domestications occurred, allowed them to determine whether to obtain the same convergent phenotypes, evolution took part in the selection of the same genomic regions or of completely different set of genes that code for the same phenotypes. Interestingly, only a small portion of the genome and of genes identified as putatively under selection during domestication were shared between the two gene pools, which suggested different genetic routes to domestication (Schmutz et al., 2014). This outcome appears to suggest that the sexually compatible Mesoamerican and Andean lineages with similar morphologies and life cycles underwent independent selection upon distinct sets of genes. However, taking into account that explicit demographic modeling was not used to generate an expectation of the number of potential false positive regions by Schmutz et al. (2014), another possible explanation for this result is that the lack of correlation between the two gene pools is due to a high level of false positives; i.e., regions of the genome with reduced diversity due to the stochastic effects of domestication bottlenecks. Regarding this consideration, Bitocchi et al. (2016a) compared their candidates for selection during domestication of common bean in Mesoamerica with those of other studies (Bellucci et al., 2014a; Schmutz et al., 2014; Rodriguez et al., 2016), and found that two (AN-Pv69, AN-DNAJ) out of the four strong candidates identified were detected as outliers by Schmutz et al. (2014) only during Andean domestication. This implies that more studies are needed either to support or refute the lack of correlations between the two gene pools found by Schmutz et al. (2014).

## Dissemination of *Phaseolus* Crop Species Outside their Centers of Origin

Beans are widely cultivated out of the Americas, and especially the common bean, which is the main *Phaseolus* crop species cultivated worldwide. For this reason, almost all the literature that has focused on investigation of the process of dissemination of beans out of their domestication centers is on *P. vulgaris*, with considerably less knowledge, if any, regarding the other *Phaseolus* crop species.

For common bean, a very complex scenario was highlighted by numerous studies, which includes: (i) several introductions from the New World, in combination with exchanges between continents, and among several countries within continents; (ii) new agro-ecological conditions experienced by this crop, implying new opportunities for both natural and human-mediated selection to act; and (iii) loss of spatial isolation characteristic of the Americas, which allowed hybridization and introgression between the Andean and Mesoamerican gene pools, and as a consequence, the occurrence of novel genotypes and phenotypes that transgressed the parental phenotypes for important agronomic and adaptive traits, such as, e.g., nutritional quality and resistance to biotic and abiotic stresses (Angioi et al., 2010; Blair et al., 2010; Gioia et al., 2013a).

### Patterns of Diversity of Beans Out of American Centers of Domestication

High levels of genetic diversity have been reported for common bean populations cultivated worldwide, and several continents and countries have been proposed as secondary centers of diversification for this species. These have included: the Iberian Peninsula (Santalla et al., 2002), the whole of Europe (Angioi et al., 2010, 2011; Gioia et al., 2013a), Brazil (Burle et al., 2010), central-eastern and southern Africa (Martin and Adams, 1987a,b; Asfaw et al., 2009; Blair et al., 2010), and China (Zhang et al., 2008).

In South America, a particular situation has emerged in Brazil. Although Brazil is closer to the Andes than to Mesoamerica, unexpectedly, it is the Mesoamerican *P. vulgaris* that is more prevalent (Burle et al., 2010). Multiple introductions of Mesoamerican germplasm in periods antecedent or successive to the discovery of the Americas might explain this pattern (Gepts et al., 1988).

In Africa, overall, the two gene pools are approximately equal in frequency, albeit there are strong differences between countries (Angioi et al., 2011; Okii et al., 2014). Such differences have been explained by the existence of at least partially independent seed networks in different countries (Asfaw et al., 2009), and because of selection due to dissimilar ecological and economic conditions among countries (Wortmann et al., 1998; Asfaw et al., 2009). Differential resistance to soil-borne diseases like *Fusarium* root rot, and different yield performances arising from the ‘interference’ of improved genotypes released by national breeding programs have also been considered to explain the uneven distribution of the two gene pools across these regions (Blair et al., 2010).

In China, a prevalence of the Mesoamerican *P. vulgaris* has been observed (Zhang et al., 2008), although this was attributed mainly to founder effects (Zhang et al., 2008). The Himalayan region, as also the entire Indian subcontinent, shows high genetic diversity (Sofi et al., 2014; Rana et al., 2015).

Little is known about the dissemination of the common bean in India, albeit trading in the 16th century via the Red Sea and the Arabian Sea, and through the Hindustan Silk route, probably had a determinant role in the dissemination of this crop. It is also possible that the sea route discovered by the Portuguese explorer, Vasco da Gama, had a role in this dissemination. The genetic diversity in India also includes the combination of both the Mesoamerican and the Andean gene pools (Rana et al., 2015). Moreover, adaptation to micro-geographic conditions has been suggested for these landraces. Indeed, the analysis of >4000 landraces allowed the identification of several diverse clusters, irrespective of the place of collection, which also indicates a strong role for gene flow.

The Mesoamerican and Andean gene pools were both introduced into Europe. Studies carried out using several different marker types have shown that the Andean *P. vulgaris* predominates over Mesoamerican *P. vulgaris* (Gepts and Bliss, 1988; Lioi, 1989; Santalla et al., 2002; Logozzo et al., 2007; Angioi et al., 2010). The Andean type is largely predominant for the Iberian peninsula, Italy and central-northern Europe, where it is also prevalent on a local scale (Sicard et al., 2005; Angioi et al., 2009b). In the eastern part of Europe, the frequency of the Mesoamerican type tends to increase but it is always lower than the Andean (Papa et al., 2006). In their study of the expansion of the common bean *P. vulgaris* in Europe, Angioi et al. (2010) concluded that the intensity of the cytoplasmic bottleneck that resulted from this introduction into Europe was very low or absent (i.e., a loss of cpSSR diversity of *∼*2%).

Regarding the other *Phaseolus* crop species, there is little in the literature that has focused on the investigation of genetic diversity of *P. coccineus*, the allogamous sister species of *P. vulgaris*, in Europe. Some studies were conducted on small (Nowosielski et al., 2002; Sicard et al., 2005; Acampora et al., 2007; Boczkowska et al., 2012) and ample (Spataro et al., 2011; Rodriguez et al., 2013) spatial scales. The introduction of *P. coccineus* into Europe was probably contemporary with that of the common bean *P. vulgaris* (Westphal, 1974). Among the Mediterranean countries, *P. coccineus* is more widespread in Spain and Italy, while in northern Europe, it occurs more often in the UK and The Netherlands, where *P. coccineus* has often substituted for *P. vulgaris* (Santalla et al., 2004) as it is more adapted to cold temperatures and cool summers than *P. vulgaris* (Delgado Salinas, 1988; Rodino et al., 2007).

However, overall, *P. coccineus* is characterized by a narrower adaptability to environmental conditions than *P. vulgaris*. As previously mentioned, *P. coccineus* and *P. vulgaris* are cross-fertile when *P. vulgaris* is the maternal parent, and this might have allowed hybridisation between these two species in Europe, where they often coexist in close sympatry in the same field. However, no evidence of introgression between common bean and runner bean has been found (Sicard et al., 2005). Data on nuclear and chloroplast variability of *P. coccineus* indicate that in Europe it has at least two main genetic groups (Spataro et al., 2011; Rodriguez et al., 2013). Of particular interest, there is a highly significant association between latitude and phenology for *P. coccineus* (Rodriguez et al., 2013). This relationship still holds when the effects of population structure for cpSSRs and nuSSRs is factored out. Therefore, this correlation is not just a consequence of the uneven geographic distribution of the two *P. coccineus* gene pools across Europe. It was then suggested that selection (probably for photoperiod sensitivity, and/or for low temperature), rather than migration and gene flow, has also had a role in shaping the population structure of *P. coccineus* in Europe (Rodriguez et al., 2013).

A comparison between Spanish and Mexican accessions of *P. vulgaris* and *P. coccineus* suggested that *P. coccineus* has maintained a high level of diversity since its introduction into Europe (Alvarez et al., 1998). A more recent study analyzed a worldwide collection of *P. coccineus*, and this analysis indicated that limited diversity of the runner bean *P. coccineus* appears to have been introduced into Europe, and that for nuclear markers, the European landraces show a reduction in diversity of 33% compared to that of the Mesoamerican landraces (Spataro et al., 2011). More recently, the use of chloroplast markers indicated a moderate-to-strong cytoplasmic bottleneck that followed the expansion of *P. coccineus* into Europe, with a reduction of 13% in chloroplast diversity (Rodriguez et al., 2013). As these markers are the same in number and type as those used by Angioi et al. (2010) to estimate the bottleneck of *P. vulgaris* following its expansion into Europe (2%), it can be concluded that the loss of diversity in *P. coccineus* appears to be stronger than in *P. vulgaris*.

Both nuclear and chloroplast analyses have shown that Mesoamerican and European *P. coccineus* accessions belong to distinct gene pools (Spataro et al., 2011; Rodriguez et al., 2013). It can be hypothesized that the differentiation of the European gene pool was due to adaptation to the new environment, and to genetic drift and a lack of introgression from wild forms.

The introduction of *P. lunatus* in Europe was very limited, with very few examples (Doria et al., 2012).

### A Role for Adaptive Introgression for the Evolution of European Beans?

Using isozyme markers, Santalla et al. (2002) estimated a high percentage of hybrids in their common bean landrace collection from the Iberian peninsula (25%). Then later, through the integration of cytoplasmic and nuclear analyses, Angioi et al. (2010) reported that about 44% of their wide collection of landraces that spanned almost all of the European countries appeared to be derived from at least one hybridisation event. In addition to the molecular results, Angioi et al. (2010) showed that seed size and coat traits vary with the level of introgression between the two gene pools. More recently, Gioia et al. (2013a) used population assignment techniques to also reveal extensive hybridisation between the two gene pools in Europe, with a frequency of hybridisation that was almost fourfold greater in Europe (40.2%) than in the Americas (12.3%), which confirmed the findings of Angioi et al. (2010). This can be explained by the geographic isolation between the gene pools in the American centers of origin, and that following the introduction into Europe, genotypes from different genes pools often coexisted on very small spatial scales (i.e., in small cultivated areas), and thus had the chance to hybridize. Estimations of hybridisation in other parts of the world have always been <10%, and so much less than in Europe (Zhang et al., 2008; Asfaw et al., 2009; Blair et al., 2010; Burle et al., 2010). Taken all together, this evidence supports the hypothesis that the whole of Europe can be regarded as a secondary center of diversity for the common bean (Angioi et al., 2010), as also suggested by the work of Santalla et al. (2002) that was limited to the Iberian peninsula.

After its introduction into Europe, *P. vulgaris* was exposed to new and ample agro-ecological conditions. Thus, it is likely that any releases that were not adapted to the new conditions were initially purged by selection. Among the natural factors, biotic and abiotic stress were probably determinants in shaping the genetic structure of the European bean landraces. It is possible that their adaptation to long days, their cold tolerance, and their resistance to pests and diseases were crucial, and this would probably have led to a reduction in the diversity that was initially present in the founding populations. Additionally, the selection operated by farmers for seed color and size, and culinary, organoleptic and nutritional quality might also have had strong impact on the evolution of the European bean, as witnessed by the myriad of local bean populations with particular characteristics and specific names (Angioi et al., 2009b; Lioi and Piergiovanni, 2013; De Ron et al., 2016). Moreover, the documented scenario of extensive Mesoamerican × Andean gene pools through their hybridisation in Europe (Logozzo et al., 2007; Angioi et al., 2010; Gioia et al., 2013a) suggests that introgressive hybridisation might have been the fundamental ‘evolutionary stimulus’ (Anderson and Stebbins, 1954; Lewontin and Birch, 1966) that propelled and boosted bean evolution in Europe. Indeed, hybridisation can produce new genotypic and phenotypic combinations that do not occur in either of the parental taxa, and upon which selection might act.

Recently, Bitocchi et al. (2015) reported that European flint landraces grown *in situ* show adaptive introgression from modern maize. A key result of their study was that adaptation followed by hybridisation has been very rapid, with landraces capturing and increasing the frequency of favorable alleles over very short times (e.g., 50 years) (Bitocchi et al., 2015). This allows the hypothesis that this evolutionary mechanism might also have operated for the European bean landraces, which have a history that is some 10-fold longer. This is of interest not only for studies in evolutionary genetics, but also for plant breeders. Indeed, studies focused on hybridisation have shown the potential for the identification of functionally important regions of the genome (Arnold and Martin, 2010).

### The Possibility of Unraveling the Architecture of Adaptation of *Phaseolus vulgaris* in Europe

The potentiality of the studies at continental scale on the architecture of adaptation in common bean is confirmed by the possibility to apply a signature of selection approach using European landraces. Indeed, using methods for the identification of outlier loci for selection, Santalla et al. (2010) provided evidence that selective forces have had significant roles, particularly for seed size, growth habits, pest resistance and flowering time. This is intriguing, as selection for flowering time was probably a key element in the history of the bean also in its center of origin.

The BEAN_ADAPT project^2^ is funded through the 2nd ERA-CAPS call, ERA-NET for Coordinating Action in Plant Sciences. The main aim of this project is to dissect out the genetic basis and phenotypic consequences of the adaptation to new environments of *P. vulgaris* and its sister species *P. coccineus*, through the study of their introduction from their respective centers of domestication in the Americas and their expansion through Europe as a recent and historically well-defined event of rapid adaptation. BEAN_ADAPT thus plans to characterize a large collection (11,500 accessions of each species) from three major genebanks by genotyping-by-sequencing. This will define the population structure and obtain subsets of genotypes for phenotyping (e.g., field, growth chamber) and for deeper genomic–transcriptomic–metabolomic characterisation. A multidisciplinary approach is planned (i.e., genomics, population/quantitative genetics, biochemistry, plant physiology) to unraveling the genetic bases of adaptation of these crops in new agro-ecological environments. The methods here rely on previous studies that have demonstrated the effectiveness of various analysis, such as the application of: (i) population genomics, to test for signatures of selection (e.g., Bellucci et al., 2014a); (ii) evolutionary metabolomics, which appear to be a very powerful approach to characterize molecular phenotypic changes due to domestication, and to identify traits under selection in wheat (Beleggia et al., 2016); (iii) association mapping studies of complex traits, such as flowering time (Raggi et al., 2014); (iv) analysis of signatures of selection by searching for ‘unusually high’ correlations between SNPs and environmental variables at the continental scale, as successfully applied by Rodriguez et al. (2016) for wild common bean from Mexico.

## Conclusion

This review presents a comprehensive overview of the current knowledge about the evolutionary history of the *Phaseolus* crop species. This takes us from the origins and evolution of their wild forms, with their co-evolution and interactions with humans and diverse environments during and after the domestication process, to their colonization of new environments out of their centers of origin and domestication. The picture that has unraveled shows that the specific and almost unique features of the *Phaseolus* genus make it a very powerful model to address important evolutionary issues. In particular, among crops, *Phaseolus* represents a unique example of multiple parallel domestications for five closely related species, with two of these (i.e., *P. vulgaris* and *P. lunatus*) each domesticated independently in Mesoamerica and the Andes. This has resulted in at least a total of seven independent domestication events, involving species that have diverged relatively recently (∼4 Ma; Delgado-Salinas et al., 2006) and that show similar genomic structures, making inter-specific comparisons feasible. This represents a ‘domestication experiment’ with full factorial design for three factors, as species, areas and wild/domesticated status, which can provide a deep understanding of the genomic architecture of domestication. Moreover, the study of different domestication events provides valuable replicates for the understanding of convergent evolution (i.e., different species or populations that evolved similar phenotypes) and its genomic determinants and effects. The possibility of considering these multiple parallel domestications as different replicates of the same experiment is also supported by the recent findings of Vlasova et al. (2016), who reported that both lineages of *P. vulgaris* and potentially all of the *Phaseolus* species share the same patterns of gene duplication that predate the divergence between the Mesoamerican and Andean gene pools.

Along with domestication, the complex patterns of dissemination of *Phaseolus* crops out of their centers of domestication represents a further key strength to unravel the genetic basis of plant adaptation. This is exactly what the BEAN_ADAPT project is searching for: to dissect out the genetic basis and phenotypic consequences of the adaptation to new environments of the common bean and its sister species, the runner bean, through the study of their introduction and expansion through Europe, as a recent and historically well-defined event of rapid adaptation.

This approach can provide a model for future major environmental and socio-economic changes, such as increases in temperature, variability of rainfall, and new consumer preferences, which will be fruitful for both evolutionary biologists and plant breeders. For the evolutionary biologists, it will be of particular interest to compare the results obtained across different species and populations, to look for patterns of convergent evolution, at either the phenotypic or molecular level. Discovering genes and genetic mechanisms that contribute to phenotypic adaptation associated with environmental conditions and their mapping along the reference genome will provide a useful genetic tool for geneticists and breeders for the design of novel varieties.

## Author Contributions

RP and EBi designed and wrote the manuscript. EBe, DS, LN, EBi, and RP contributed to the drafting of the manuscript especially for the sections on the origin and domestication of *Phaseolus* ssp., while DR, MM, MR, GA and TG contributed to the drafting of the manuscript especially for the sections on dissemination out of the domestication centres of *Phaseolus* ssp.

## Conflict of Interest Statement

The authors declare that the research was conducted in the absence of any commercial or financial relationships that could be construed as a potential conflict of interest.

## Footnotes

1 http://www.diva-gis.org/

2 http://www.beanadapt.org/
