# Supplementary material for: Beans (Phaseolus ssp.) as a Model for Understanding Crop Evolution
Source: Front Plant Sci. 2017 May 8;8:722. doi: 10.3389/fpls.2017.00722 (PMC5420584; doi:10.3389/fpls.2017.00722)
Supplement: Supplementary file 1 [file Table_1.DOCX]

**Table S1.** Ecological variables for each of the five *Phaseolus* crop species.

| **Ecological variable** | ***P. vulgaris*** | ***P. coccineus*** | ***P. dumosus*** | ***P. acutifolius*** | ***P. lunatus*** |
| --- | --- | --- | --- | --- | --- |
| **N° accessions** | 410 | 125 | 9 | 82 | 170 |
| **ALT** | 1,804.4 ± 670.0 | 2,085.2 ± 513.7 | 1,828.1 ± 361.9 | 1,392.4 ± 678.7 | 1,168.4 ± 774.4 |
| **BIO1** | 18.0 ± 3.6 | 16.2 ± 3.1 | 17.5 ± 2.3 | 18.5 ± 3.6 | 21.2 ± 4.0 |
| **BIO2** | 13.5 ± 2.6 | 13.6 ± 2.5 | 10.8 ± 0.6 | 16.4 ± 1.7 | 11.3 ± 2.0 |
| **BIO3** | 69.5 ± 9.6 | 70.1 ± 3.9 | 73.7 ± 2.0 | 55.7 ± 6.1 | 76.8 ± 7.7 |
| **BIO4** | 188.1 ± 123.5 | 159.4 ± 74.9 | 95.0 ± 15.9 | 497.3 ± 149.8 | 99.0 ± 70.5 |
| **BIO5** | 27.7 ± 4.5 | 25.7 ± 3.8 | 24.5 ± 2.0 | 33.2 ± 3.4 | 28.6 ± 4.8 |
| **BIO6** | 7.7 ± 5.0 | 6.2 ± 4.0 | 9.9 ± 3.0 | 3.4 ± 4.3 | 13.6 ± 3.8 |
| **BIO7** | 20.1 ± 5.4 | 19.5 ± 4.3 | 14.6 ± 1.0 | 29.7 ± 4.1 | 15.0 ± 3.5 |
| **BIO8** | 19.3 ± 3.5 | 17.3 ± 3.1 | 18.1 ± 2.1 | 23.5 ± 3.8 | 21.6 ± 4.3 |
| **BIO9** | 16.7 ± 4.4 | 14.8 ± 3.4 | 16.5 ± 2.7 | 18.4 ± 3.8 | 20.5 ± 4.0 |
| **BIO10** | 20.1 ± 3.7 | 18.0 ± 3.2 | 18.5 ± 2.2 | 24.3 ± 3.6 | 22.3 ± 4.4 |
| **BIO11** | 15.5 ± 4.2 | 14.1 ± 3.2 | 16.2 ± 2.5 | 12.3 ± 4.4 | 19.9 ± 3.8 |
| **BIO12** | 1,111.3 ± 538.2 | 1,205.3 ± 457.6 | 1,634.0 ± 432.3 | 554.4 ± 208.6 | 1,566.0 ± 684.4 |
| **BIO13** | 220.9 ± 82.7 | 246.5 ± 87.0 | 346.6 ± 71.8 | 136.3 ± 53.9 | 292.3 ± 115.3 |
| **BIO14** | 12.3 ± 18.2 | 9.4 ± 8.2 | 4.9 ± 3.7 | 5.2 ± 3.9 | 17.1 ± 15.4 |
| **BIO15** | 93.0 ± 24.0 | 94.1 ± 11.0 | 97.1 ± 5.2 | 99.5 ± 18.7 | 80.5 ± 20.9 |
| **BIO16** | 589.2 ± 218.4 | 635.6 ± 206.8 | 824.0 ± 179.2 | 347.3 ± 143.2 | 750.1 ± 295.5 |
| **BIO17** | 49.8 ± 67.9 | 36.2 ± 27.0 | 22.9 ± 15.3 | 23.4 ± 13.9 | 67.6 ± 56.8 |
| **BIO18** | 344.9 ± 130.5 | 402.1 ± 182.4 | 512.0 ± 205.6 | 255.3 ± 111.9 | 382.2 ± 171.5 |
| **BIO19** | 124.9 ± 201.7 | 46.7 ± 35.9 | 34.0 ± 22.0 | 69.2 ± 40.1 | 215.9 ± 244.9 |

Data are means ±SD. Geographical coordinates of the collection sites of the wild accessions with passport data present in the database of the International Centre for Tropical Agriculture (CIAT) for each of the five *Phaseolus* crop species were used to extract the ecological data using DIVA-GIS 7.5 (<http://www.diva-gis.org/>). Twenty ecological variables were considered: **ALT**, altitude; **BIO1**, annual mean temperature; **BIO2**, mean diurnal range (mean of monthly [max temp - min temp]); **BIO3**, Isothermality (BIO2/BIO7) (* 100); **BIO4**, temperature seasonality (standard deviation *100); **BIO5**, maximum temperature of warmest month; **BIO6**, minimum temperature of coldest month; **BIO7**, temperature annual range (BIO5-BIO6); **BIO8**, mean temperature of wettest quarter; **BIO9**, mean temperature of driest quarter; **BIO10**, mean temperature of warmest quarter; **BIO11**, mean temperature of coldest quarter; **BIO12**, annual precipitation; **BIO13**, precipitation of wettest month; **BIO14**, precipitation of driest month; **BIO15**, precipitation seasonality (coefficient of variation); **BIO16**, precipitation of wettest quarter; **BIO17**, precipitation of driest quarter; **BIO18**, precipitation of warmest quarter; **BIO19**, precipitation of coldest quarter.
